# Motives and attitudes of parents toward HPV vaccination: Results from the initial period of HPV vaccine rollout in Serbia

**DOI:** 10.1371/journal.pone.0287295

**Published:** 2023-07-06

**Authors:** Mirjana Štrbac, Vladimir Vuković, Tatjana Pustahija, Nataša Nikolić, Smiljana Rajčević, Svetlana Ilić, Tihomir Dugandžija, Aleksandra Patić, Mioljub Ristić, Vladimir Petrović

**Affiliations:** 1 Institute of Public Health of Vojvodina, Novi Sad, Serbia; 2 Faculty of Medicine, University of Novi Sad, Novi Sad, Serbia; 3 Oncology Institute of Vojvodina, Sremska Kamenica, Serbia; Carol Davila University of Medicine and Pharmacy, ROMANIA

## Abstract

Human papillomavirus (HPV) infection is one of the most common viral infections in sexually active population worldwide, and is the main cause of cervical cancer, which is the fourth most common cancer among women. Serbia ranks third in incidence and mortality rate of cervical cancer in Europe. We conducted a cross-sectional study considering parents’ motivation for the HPV vaccination of their children. Statistical analysis included descriptive statistics and a logistic regression model. We found that the strongest motive was “Recommendation from paediatrician” (20.2%), followed by the attitude that HPV vaccine protects against cancers in different localization (15.4%), the motive “It is better to vaccinate a child than expose them to potential risk of HPV infection” (13.3%) and “Feeling anxiety due to a possible infection and cancer in the child” (13.1%). For those parents that vaccinated their child for some other strongest motive, reasons like “Vaccine is free of charge”, “Recommendation from friends and family” and motive „My child received all obligatory vaccines, so I want to receive this one as well“, were significantly more frequently selected. In the group where paediatricians’ recommendation was not a motive for accepting the HPV vaccine, the largest percentage of parents (89.6%) selected motive “HPV vaccine protects against cancers in different localization” and the motive “It is better to vaccinate a child than expose them to potential risk of HPV infection” (78.1%). Paediatrician’s recommendation is very important for parents’ decision to vaccinate, however, other motives also influenced and had significance in making the parents’ decision to vaccinate their children against HPV. Encouraging trust in public health authorities in Serbia, highlighting the advantages of the HPV vaccine and further encouraging healthcare workers to give stronger recommendations can increase the HPV vaccine uptake. Finally, we provided the basis to create more targeted messages that will empower parents to vaccinate their children.

## Background

Human papillomavirus (HPV) infection is one of the most common viral infections in sexually active population, although the majority of HPV infections are asymptomatic and resolve spontaneously, persistent infection with HPV may result in disease [1]. Cervical cancer is the fourth most common cancer among women, with an estimated 604,127 new cases and 341,831 deaths in 2020 worldwide [2], and 12% of estimated HPV prevalence (all types) among adult women with normal cytological findings. Current estimates show that the population of Serbia ranks third, in both, the incidence rate and the mortality rate of cervical cancer, in respect to the rest of Europe [2]. Significant fact is that, in a population of 3.82 million women in Serbia, more than 1205 women annually get cervical cancer and about 634 women die from this disease [3]. Previous published data showed that the prevalence of HPV type 16/18 positivity is as high as 31.6% on a routine cytological cervical cancer screening in a sample of Serbian women and it is estimated that about 90% of the population become infected with HPV at some point during their lifetime [4]. Also, according to published data, the frequency of genital warts caused by HPV types 6 and 11 is significantly higher in the age group of 15 to 19 years old compared to other age groups in Novi Sad [5]. Considering the fact that more than 90% of cervical cancer cases are caused by the HPV infection, it was demonstrated that by preventing the infection we also prevent the occurrence of cervical cancer, as well as, cancer of other anogenital localization and head and neck cancers in both men and women [6,7].

Vaccination against the HPV is one of the most effective ways of HPV-related cancer prevention, especially when it is administered to adolescents before the initiation of sexual activity [8]. In particular, the impact of HPV vaccination in real life conditions has become evident among women who are vaccinated before the exposure to HPV, especially in countries with high vaccine coverage [9]. Currently, six prophylactic HPV vaccines are licensed. All HPV vaccines contain VLPs against high-risk HPV types 16 and 18; the nonavalent vaccine also protect against high-risk HPV types 31, 33, 45, 52 and 58; and the quadrivalent (HPVq) and nonavalent vaccines contain VLPs to protect against anogenital warts causally related to HPV types 6 and 11 [1]. The HPV vaccine provides protection against anogenital warts for 80–100% vaccinees and can reduce new cases of pre-malignant lesions in 60–80% vaccinees [10–14]. The HPV vaccines can be administered as early as 9 years of age, while recommended catch-up vaccination includes females up until 26 years [1].

Since 2008, in Serbia, the HPV vaccine has been recommended for children, before the first sexual intercourse, but for those first available HPV vaccine, the health insurance did not cover its cost [15]. In 2020, the Institute of Public Health of Vojvodina (IPHV), Novi Sad (Serbia) introduced a local promotional campaign of HPV immunization, where vaccine was free of charge, for girls from the city of Novi Sad and, in 2021, the promotional campaign was extended to include boys as well (gender neutral programme). This campaign had a specific goal to raise awareness and knowledge about the significance of HPV vaccine and to offer this vaccine, free of charge, before introducing it into the National Immunization Program (NIP) in Serbia. Paediatricians from the Primary Healthcare Centre (PHC) of Novi Sad, who are responsible for providing vaccines during the paediatric age, those obligatory vaccines included in the NIP as well as those recommended per certain age, were additionally trained about the HPV vaccine, its method of application, contraindications, as well as, about how to offer vaccine to teenagers 12–18 years old. Also, they were encouraged to contact the competent epidemiologist from the IPHV in case that they had additional questions during their communication with parents. At the end of the promotional campaign (2020–21), about 1400 teenagers were vaccinated with two or three doses of the HPV vaccine, depending on their age. Thanks to the great experience during the local promotion campaign of the HPV vaccine in Novi Sad, later in 2022, nine-valent HPV vaccine was introduced in the NIP in Serbia and applied across the country [15].

Even though there is plenty of evidence about the fact that development of the HPV vaccine goes along with a new era in cancer prevention, the HPV vaccination programs for children have caused a great deal of controversy among the public, including parents [16]. A variety of parents’ reasons for rejected HPV vaccine have been described, including: a lack of research on long-term efficacy and side effects of the vaccine, age of vaccination that is too young, mistrusting the pharmaceutical companies, and fear that vaccines would promote early sexual activity in vaccinated children [17].

The HPV vaccination program in Serbia is focused mainly on young adolescents whose parents gave consent on vaccination, so the success of the HPV vaccination program depends on the parents’ motivation and their decision. Several studies have demonstrated that healthcare providers are the most valuable and trusted source of information for parents regarding their decision whether to vaccinate their children [18,19]. Additionally, physicians’ knowledge and attitudes toward vaccination can significantly influence the decision regarding HPV vaccination in parents [20].

Parents’ attitudes could be the main barrier to HPV immunization compliance. So, it is very important to assess parental knowledge and attitudes towards this preventive strategy before organization and implementation of the National HPV immunization program in Serbia [21].

Still, there is a lack of published data about the motivation(s) of Serbian parents who already accepted HPV vaccination of their children, when the HPV vaccine became widely available in Serbia. The aim of this study was to recognize the main motives for accepting the HPV vaccination by parents of the eligible children, to determine the strongest, as well as, the most prevalent parent’s motive(s) for accepting the HPV vaccine. In addition, this study aimed to compare general characteristics and motives of parents who selected paediatrician’s recommendation as their strongest motive in comparison with those who were not. We also aimed to determine potential predictors for parents to select other motives for HPV vaccination rather than the paediatrician’s recommendation as an important motive. These results might be crucial for implementation of the additional HPV-vaccination promotional campaigns at the start of the implementation of the National HPV immunization program in Serbia, in the 2022 year.

## Methods

We conducted a cross-sectional survey in the pediatric’s department of the PHC Novi Sad, from January to December 2021 in the city of Novi Sad, using a structured questionnaire which consisted of 17 motives and attitudes that could have served as a potential driver for parents’ consent for HPV vaccination of their child(ren). We recruited parents/legal guardians of children aged 12–18 years. A total of 676 parents/legal guardians were invited and 436 agreed to participate in the study (response rate 64.5%). The questionnaire was anonymous and it was fulfilled by the parents/legal guardians prior to the HPV vaccination of their children, in the PHC of Novi Sad. Study questionnaire comprised of two segments: 1) parents’ sociodemographic characteristics, as well as, age and gender and number of their children, and 2) question with 17 offered motives and attitudes to acceptance of the HPV vaccination (for each of the 17 motives, parents were signed the most important motive for HPV vaccination of their child).

The study protocol was approved by the Ethics Committee of the PHC Novi Sad under the number 21/21-1. Written consent was obtained from each participant.

For the purpose of the analysis, we divided the participants into two groups based on their response to the motive “Paediatrician’s recommendation” to those that signed this motive as more important for accepting HPV vaccine in comparison with others. Further, we compared characteristics and additional motives of those parents.

The HPV vaccine was offered free of charge to the residents of the city of Novi Sad during this promotional campaign, a year before implementation of the nonavalent HPV vaccine in Serbia. Parents were informed through the local media and during health visits to the paediatric’s department about the possibility to bring their child(ren) to the PHC of Novi Sad where they can be immunized with the HPV vaccine.

Descriptive statistics was used with categorical variables that are presented as absolute frequencies and percentages (%) while the continuous data are presented as mean with standard deviation (SD). Chi-squared test or Fisher’s exact test (in case when only few observations for individual cells were reported) for categorical variables, and Wilcoxon rank-sum test and t-test for discrete variables were performed. In order to determine the strongest motive for the HPV vaccination among parents who did not select paediatrician recommendation as the main motive for vaccination, univariable and corrected multivariable logistic regression analyses were performed. All statistical analyses of the data were performed using statistical software package Stata v.16 (College Station, TX: StataCorp LLC. 2019). The statistical significance was set at p < 0.05.

## Results

A total of 436 (64.5%) parents of the 676 vaccinated children during the study period, were included, and 240 (35.5%) parents did not agree to participate in this study. The mean age of parents was 45.1 years (SD = 4.2) and 82.6% were female. Majority of the participants (53.9%) had high secondary level of education and 83.9% of all respondents were married. Further, 96.1% of families had at least one female child in the household. Out of the total number of included vaccinated children, most of them were 15–18 years old (66.7%), with the mean age of 14.3 years (SD = 1.6), and 90.8% were girls (Table 1).

**Table 1:** Demographic characteristics of study participants (n = 436) and their children.

Variable	Number	Percentage (%)
Parent’s age, mean ± SD	45.1±4.2	
Gender of parents		
Mother	360	82.6
Father	76	17.4
Parent’s level of education—school		
Elementary	8	1.8
Secondary	235	53.9
Faculty	193	44.3
Parent’s marital status		
Single	70	16.1
Married	366	83.9
Number of female children in the household		
None	17	3.9
One	319	73.2
Two and more	100	22.9
Number of male children in the household		
None	297	68.1
One	130	29.8
Two and more	9	2.1
Age of the vaccinated child, mean ± SD	14.3 ± 1.6	
Age category of the vaccinated child		
12–14 years	145	33.3
15–18 years	291	66.7
Gender of the vaccinated child		
Girl	396	90.8
Boy	40	9.2

**Fig 1:**
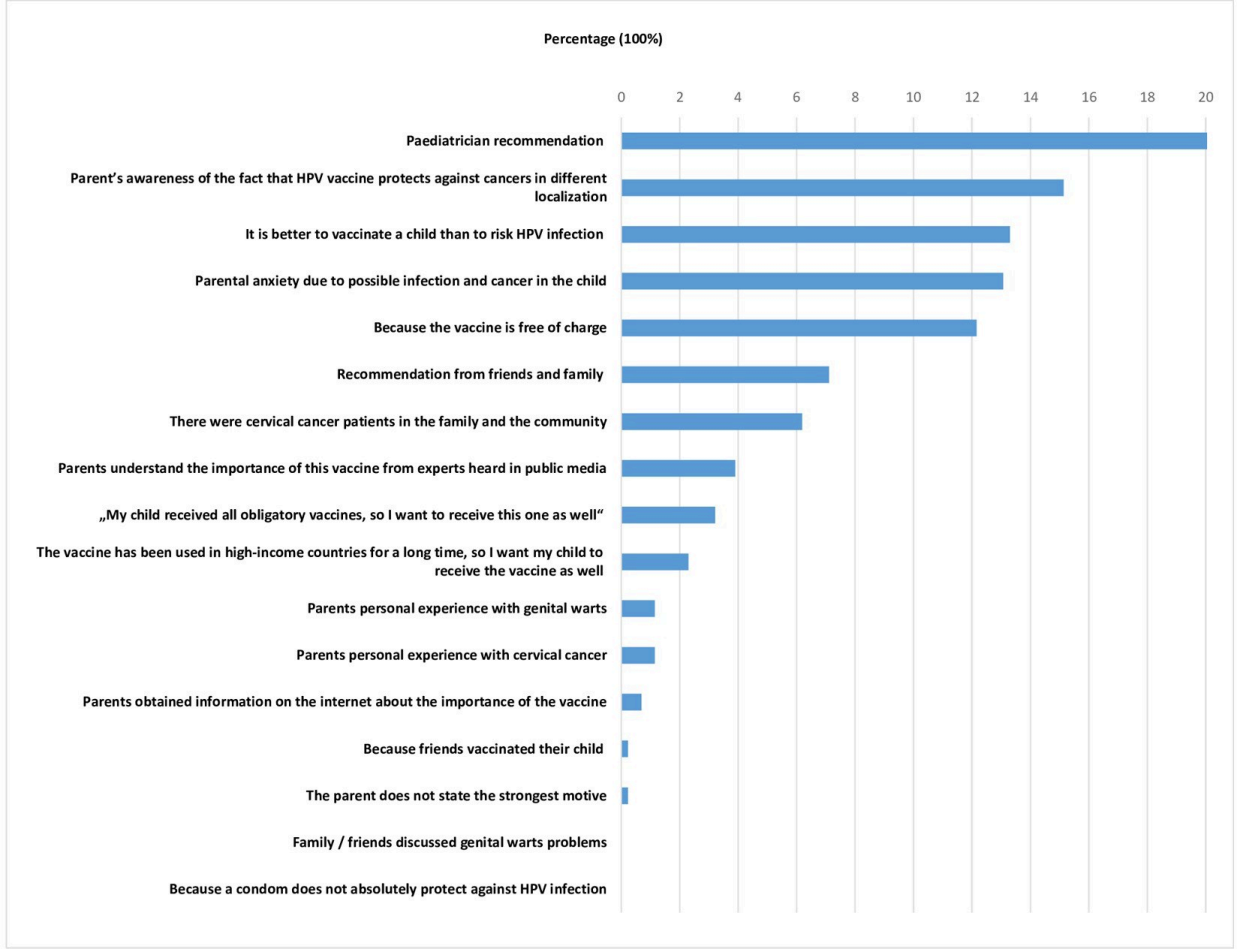
The strongest motive for accepting the HPV vaccination among parents of the vaccinated children.

Among the 17 offered motives and attitudes, the most frequently selected motive (20.2%) as the strongest for accepting the HPV vaccination was the “Recommendation from paediatrician”, as presented in the Fig 1. A considerable number of parents (15.4%) were motivated to vaccinate their child(ren) based on the awareness that HPV vaccine protects against cancers in different localization. In addition, some of the participants (13.3%) were pointed to the attitude that “It is better to vaccinate a child than potentially exposure them to the risk of HPV infection”, and others (13.1%) answered that they vaccinated children due to “Anxiety due to a possible infection and cancer in the child”. For 12% of included parents, the strongest motive for vaccination was the “HPV vaccine was offered free of charge”.

When we analysed characteristics of the participants based on their strongest motive for accepting the HPV vaccine, i.e., comparing answers for those that selected paediatrician’s recommendation (n = 88) and those who did not (n = 348), there was a significantly higher percent of children (36.6%) in the younger age category (12–14 years) among parents that selected some other motive as the strongest, in respect to the group (23.9%) that selected motive of the paediatricians’ recommendations (p = 0.04). There was no statistically significant difference when exploring other characteristics (Table 2).

**Table 2:** Characteristics of participants based on their strongest motive for accepting the HPV vaccination.

Variable	Pediatrician’s recommendation as the strongest motive (n = 88), n (%)	Any other answer as the strongest motive (n = 348), n (%)	p-value^1^
Parent’s age, mean ± SD	45.1 ± 4.2	45.1 ± 4.3	0.79
Gender of parents			
Mother	67 (76.1)	293 (84.2)	0.08
Father	21 (23.9)	55 (15.8)	
Parent’s education category			
Elementary	0	8 (2.3)	0.44
Secondary	50 (56.8)	185 (53.2)	
Faculty	38 (43.2)	155 (44.5)	
Parent’s marital status	** **		
Single	16 (18.2)	54 (15.5)	0.54
Married	72 (81.8)	294 (84.5)	
Number of female children in the household			
None	3 (3.4)	14 (4.0)	0.54
One	61 (69.3)	258 (74.1)	
Two and more	24 (27.3)	76 (21.9)	
Number of male children in the household			
None	55 (62.5)	242 (69.5)	0.42
One	31 (35.2)	99 (28.5)	
Two and more	2 (2.3)	7 (2.0)	
Age of the vaccinated child, mean ± SD	14.6 ± 1.5	14.3 ± 1.7	0.18
Age category of the vaccinated child			**0.04**
12–14 years	21 (23.9)	124 (35.6)	
15–18 years	67 (76.1)	224 (64.4)	
Gender of the vaccinated child			0.43
Girl	78 (88.6)	318 (91.4)	
Boy	10 (11.4)	30 (8.6)	

¹Indicators of significance between groups using Pearson’s chi-squared test and Fisher’s exact test (where appropriate) for categorical and Wilcoxon rank-sum test and t-test for discrete variables. Significance levels are given in bold for p<0.05.

When we compared differences in other selected motives and attitudes between 1) group of parents who selected paediatrician’s recommendation as the strongest motive, and 2) others who signed other motives as the strongest, we noticed that three motives, i.e., “Vaccine is free of charge”, “Recommendation from friends and family” as well as „My child received all obligatory vaccines, so I want to receive this one as well”were statistically significant (p<0.05) and more frequently selected in the second group (Table 3).

**Table 3:** Motives and attitudes toward accepting the HPV vaccination in two comparative groups (selection based on their strongest motive for vaccination).

Motives for HPV vaccination	Total (n = 436), n (%)	Pediatrician’s recommendation as the strongest motive (n = 88), n (%)	Any other answer as the strongest motive (n = 348), n (%)	p-value^1^
Parents personal experience with genital warts	26 (6.0)	2 (2.3)	24 (6.9)	0.13
Family / friends discussed genital warts problems	19 (4.4)	4 (4.5)	15 (4.3)	0.99
Parents personal experience with cervical cancer	4 (0.9)	0	4 (1.1)	0.59
There were cervical cancer patients in the family and the community	68 (15.6)	10 (11.4)	58 (16.7)	0.22
Parent’s awareness of the fact that HPV vaccine protects against cancers in different localization	401 (92.0)	80 (90.9)	321 (92.2)	0.68
Parental anxiety due to possible infection and cancer in the child	315 (72.2)	64 (72.7)	251 (72.1)	0.91
It is better to vaccinate a child than exposure them to potential risk of HPV infection	371 (85.1)	75 (85.2)	296 (85.1)	0.97
The vaccine has been used in high-income countries for a long time, so I want my child to receive the vaccine as well	259 (59.4)	47 (53.4)	212 (60.9)	0.20
Because the vaccine is free of charge	105 (24.1)	9 (10.2)	96 (27.6)	**<0.01**
Recommendation from friends and family	209 (47.9)	29 (33.0)	180 (51.7)	**<0.01**
Parents understand the importance of this vaccine from experts heard in public media	254 (58.3)	52 (59.1)	202 (58.0)	0.86
Parents obtained information on the internet about the importance of the vaccine	193 (44.3)	33 (37.5)	160 (46.0)	0.15
Because the condom does not absolutely protect against HPV infection	123 (28.2)	26 (29.5)	97 (27.9)	0.76
Because friends vaccinated their child	60 (13.8)	8 (9.1)	52 (14.9)	0.16
„My child received all obligatory vaccines, so I want to receive this one as well”	187 (42.9)	29 (33.0)	158 (45.4)	**0.04**
The parent does not state the strongest motive	16 (3.7)	4 (4.5)	12 (3.4)	0.54

^1^Significance levels are given in bold for p<0.05.

In our sample, a total of 183 (42%) participants responded that paediatricians’ recommendation was not relevant motive for accepting the HPV vaccine. When we compared other selected motives in this group with the rest of the participants, i.e., that selected paediatricians’ recommendation as an important motive, we noticed a significant difference in nine motives: “Parental anxiety due to possible infection and cancer in the child”; “It is better to vaccinate a child than exposure them to potential risk of HPV infection”; “The vaccine has been used in high-income countries for a long time, so I want my child to receive the vaccine as well”; “Recommendation from friends and family”; “Parents understand the importance of this vaccine from experts heard in public media”; “Parents obtained information on the internet about the importance of the vaccine”; “Because the condom does not absolutely protect against HPV infection"; „My child received all obligatory vaccines, so I want to receive this one as well“, and the category where the parent does not state the strongest motive, with the higher percentage in the group with selected Pediatricians’ recommendation (S1 Table).

**Fig 2:**
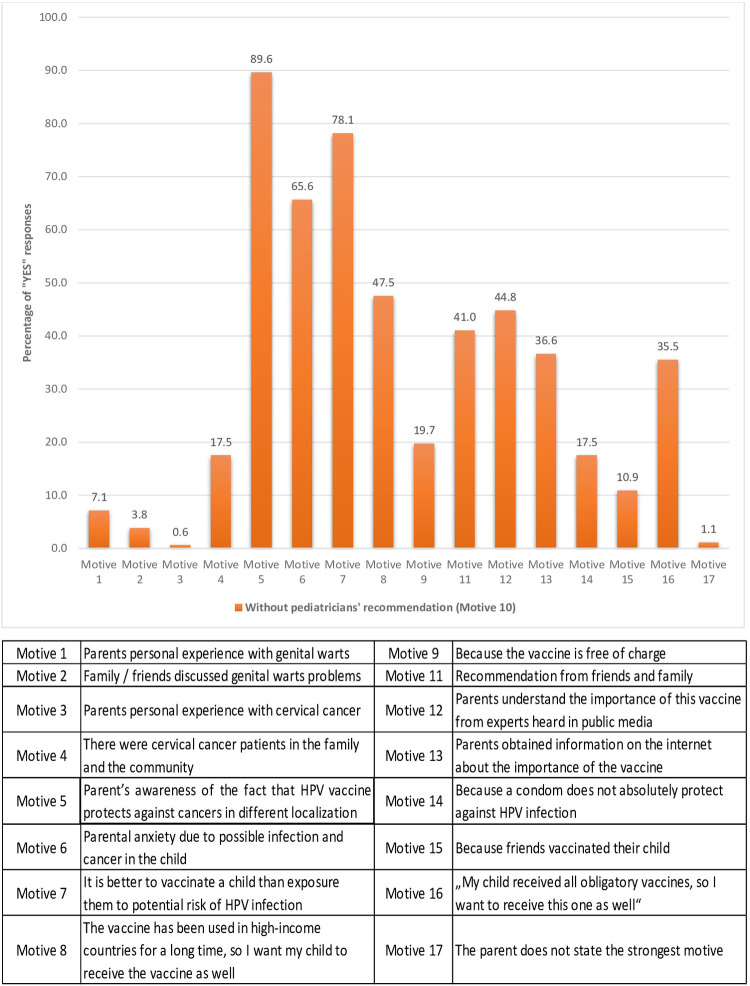
Motives for accepting the HPV vaccination in the group of parents without selecting the paediatricians’ recommendation as the most important motive.

We additionally explored motives in the group of participants who did not select paediatricians’ recommendation as the most important motive for accepting the HPV vaccine (Fig 2). The largest percentage of parents (89.6%) selected Motive 5 (Parent’s awareness of the fact that HPV vaccine protects against cancers in different localization) and a substantial portion in this group of parents was encouraged by the attitude that it is better to vaccinate a child than expose them to the potential risk of HPV infection, and also by fear and anxiety due to possible infection in the child (78.1% and 65.6%, respectively).

In order to explore the potential predictors for participants to select other motive rather than selecting the paediatrician’s recommendation as the strongest motive for HPV vaccination, univariate and multivariate analyses were undertaken. Parents of the children aged 12–14 years old had 1.77 times the odds to selecting one of these motives as the strongest compared to the parents of children in the older age category (OR = 1.77, 95% CI: 1.03–3.02, p = 0.04). The effect was even more prominent when adjusting the analysis for age and gender of the parent (aOR = 1.85, 95% CI: 1.07–3.18, p = 0.03) (Table 4).

**Table 4:** Predictors of selection of the strongest motive for the HPV vaccination other than the “Paediatrician’s recommendation”.

Variable	Crude OR (95% CI)	p-value	Adjusted OR (95% CI)¹	p-value
**Parent’s age, years**	1.00 (0.95–1.06)	0.99	-	-
**Gender of parents**				
Mother	ref.		-	-
Father	0.60 (0.34–1.06)	0.08	-	-
**Parent’s age category**				
30–39	ref.		-	-
40–49	0.69 (0.26–1.86)	0.47	-	-
50–59	0.67 (0.22–2.02)	0.47	-	-
**Parent’s level of education—school, n (%)**				
Elementary	NA		NA	
Secondary	ref.		ref.	
Faculty	1.10 (0.69–1.77)	0.69	1.04 (0.64–1.69)	0.86
**Parent’s marital status**				
Single	ref.		ref.	
Married	1.21 (0.65–2.24)	0.54	1.20 (0.65–2.23)	0.56
**Number of female children in the household**				
None	ref.		ref.	
One	0.91 (0.25–3.25)	0.88	0.85 (0.23–3.08)	0.80
Two and more	0.68 (0.18–2.56)	0.57	0.67 (0.18–2.57)	0.57
**Number of male children in the household**				
None	ref.		ref.	
One	0.73 (0.44–1.19)	0.21	0.72 (0.44–1.19)	0.20
Two and more	0.80 (0.16–3.39)	0.78	0.68 (0.14–3.44)	0.65
**Age of the vaccinated child**	0.90 (0.78–1.05)	0.18	0.89 (0.77–1.04)	0.13
**Age category of the vaccinated child**				
12–14 years	**1.77 (1.03–3.02)**	**0.04**	**1.85 (1.07–3.18)**	**0.03**
15–18 years	ref.		ref.	
**Gender of the vaccinated child**				
Girl	ref.		ref.	
Boy	0.74 (0.35–1.57)	0.43	0.75 (0.35–1.61)	0.46

¹adjusted for age and gender of the parent.

## Discussion

It is a known fact that the HPV vaccination uptake rate (at least one dose) varied significantly among countries, ranging from 2.4% to 94.4%. In line with this, results of systematic review from 2017 showed that Scotland had the highest uptake, while Hong Kong had the lowest, at 2.4% to 9.1% [22]. Like the study from Greece, many studies aimed to investigate knowledge, perceptions and practices of parents toward HPV vaccination, and determine which factors are associated with parents’ decision to vaccinate their daughters [23]. This was the first cross-sectional study carried out in Serbia considering parents’ motivation and attitudes for the HPV vaccination, when the HPV vaccine became available, free of charge. It focused on the parents’ motives associated with HPV vaccination in order to carry out interventions and promotional campaigns that are more effective to increase vaccination rates.

We found that the strongest motive for HPV vaccination was the “Recommendation from paediatrician” (20.2%), followed by the motivation to vaccinate their child(ren) based on the awareness that HPV vaccine protects against cancers in different localization (15.4%), the fact that it is better to vaccinate a child than exposure them to potential risk of HPV infection (13.30%), and because they are anxious due to a possible infection and cancer in the child (13.07%). Many studies have confirmed that a paediatrician’s recommendation is the most important predictor of HPV immunization [24]. As Navarro-Illana P. et al. concluded in their study, the main factor associated with HPV vaccination was the advice of health professionals and that the most effective measures to improve vaccination coverage should focus on this profession [25].

Additionally, there was a higher percent of children in the younger age category (12–14 years) in those that selected some other motive as the strongest (35.6%) in respect to the group that selected motive paediatricians’ recommendations (23.9%). A possible reason for recommending vaccination by paediatricians in older children is their little experience with HPV-related diseases, and the consequent reduction in the perception of benefits of HPV immunization before entering into the first sexual relations [26]. Additionally, in the other group of parents that selected “Paediatric recommendation”, there were more children 14–18 years old, which is the group of teenagers, that is closer to sexual experience and perhaps the paediatrician recognised the need to present this vaccine to those parents as a primary preventive measure against the HPV infection. The HPV vaccine was a new preventive measure that paediatricians were able to use and it is possible that they only see the risk of infection in older teenagers, similarly as Kimko underlined in his paper about paediatricians’ attitudes [27]. Finally, parents of children 12–14 years old had higher odds of selecting one of the other motives or attitudes rather than paediatricians’ recommendations as the strongest in respect to parents of children in the older age group (15–18 years). We found that girls were nine times more vaccinated than boys. In the literature, such findings are common, but the difference between vaccinated girls and boys in our study is much larger compared to data from other countries. In many countries, the vaccine has not yet been approved for boys at all, and countries that vaccinate boys approved the vaccine for males much later, which may be another reason for the low number of vaccinated boys [28].

Considering the evidence that the strong recommendations from health care professionals can increase the uptake of HPV vaccines by as much as 9%, their education is essential to increase vaccination rate [29]. A study conducted in the U.S. states that distrust of health care professionals, in general, is associated with less favourable attitudes and less acceptance of any vaccines [30].

As our research was conducted when the HPV vaccine was part of promotional campaign for improvement of reproductive health of teenagers and when citizens did not have the opportunity to vaccinate their children with free of charge HPV vaccine, it turned out that the most important motive was the paediatrician’s recommendation as well as the knowledge about the vaccine and its beneficial effects that the parents previously had. Results of a study which points out that parental decision were mainly shaped by the perceived advantages of the vaccine, and together with the recommendation of the chosen paediatrician are in line with our results [28].

Motive “vaccine was free of charge” was on the fifth place, but very important as separate motive for 12.2% of parents and it is in line with Newman’s meta-analysis, suggesting that health insurance coverage of the HPV vaccination or lower cost of vaccine to parents were significant factors for vaccination [31]. Other motives that were offered to parents in the questionnaire were chosen less likely as the strongest motive for HPV vaccine, but on the individual level they should not be ignored when talking about the goal of raising vaccine uptake.

As Sitaresmi M.N. et al. proved, there was a significant correlation between increasing HPV vaccine acceptability with the improvement of awareness, knowledge, perception toward HPV infection, cervical cancer and HPV vaccination (r  =  0.32 to 0.53, p<0.001) and other numerous studies have confirmed, and we can see from valuable motives in our study, it is very important to educate parents and distribute important facts about the benefits of this vaccine and the HPV prevention [32–34]. Consequently, in this way, educated parents with sufficient knowledge will be empowered enough to ask for vaccination of their child(ren) even without a “paediatrician’s recommendation”.

Parents who chose motive 7 (It is better to vaccinate a child than exposure them to potential risk of HPV infection) are parents who seem to know the benefit of vaccine against HPV. This is consistent with other researches that highlight the importance of parents’ knowledge and positive attitude towards HPV vaccination, because they are the most important source of information about vaccines for their children [35,36]. A survey of parents shown in a study conducted in the U.S. indicated that the main reason for parents’ indecision to vaccinate their child with the HPV vaccine was precisely the lack of their own knowledge about HPV vaccine [37]. Parents were free to select more than one motive or attitude, beside the strongest, from 17 that were offered as motives, which they consider important for their decision toward HPV vaccine. Statistically significant difference was noticed in three motives, first the “Vaccine was offered to parents free of charge”,”Recommendation from friends and family”, and “My child received all obligatory vaccines, so I want to receive this one as well”. In a population-based study by Dahlstrom et al., conducted among 13,946 parents of children 12–15 years old in Sweden, the correlates of attitudes towards HPV vaccination were explored, and was concluded that vaccine safety and effectiveness are particularly important in this group of parents for the acceptance of the HPV vaccine and successfulness of the vaccination program [38].

Parents education and importance is emphasized by research and studies conducted in other countries as well [39]. This is particularly important since additional efforts by other public health experts should be focused on this group of parents, outside of the paediatrician’s office, like television, magazines and social media, in order to empower them to bring positive decision toward HPV vaccination of their children [40].

There are several strengths and limitations of our study that should be considered. Based on the observational design of this study, we were unable to assess causal-effect relationship, and this is the first limitation of our study. Second, our sample size was relatively small, even though the response rate was over 64% in our study and it included the majority of parents of vaccinated children in Novi Sad during 2021. Third, our questionnaire was not validated before its implementation in this study. Fourth, we used self-administered questionnaire that can lead to bias in answering as well as willingness to participate of those parents that are more interested and informed on the topic. Fifth, this study was conducted from January until December 2021, thus our results should be interpreted in this context since this period during the COVID-19 pandemic might have influenced population’s perception of immunisation process in general. Our study included only parents that decided to vaccinate their child(ren), thus we did not have representatives of those that were hesitant or refused vaccination and therefore our findings cannot be generalized to the entire population. Finally, we cannot exclude the possibility that some participants misunderstood some of the questions, even though the questionnaire was fulfilled in paediatricians’ office so participants were able to ask for clarification. Despite the above-mentioned potential limitations, we presume that they did not substantially compromise the main results of our study.

In conclusion, paediatrician’s recommendation is very important for the parents’ decision to vaccinate their children, highlighting good trust and collaboration with the healthcare providers, however, other motives presented in the results of this study also influenced and certainly had significance in making the parents’ decision to vaccinate their children against HPV. For example, results of previous study conducted in Serbia showed that women undergoing cervical cancer screening had moderate and low awareness of the HPV vaccine [41]. By identifying the strongest motives that improved parents’ decision to vaccinate their children against HPV, we provided the basis to create more targeted messages that will help parents decide to vaccinate their children. Additionally, a useful approach might be further encouraging trust in public health authorities in Serbia with stronger and targeted vaccine campaigns, highlighting the advantages of the vaccine, solving gaps in knowledge in healthcare providers, and encouraging paediatricians, as well as, gynaecologists [42] to give strong(er) recommendations, which can lead to increasing the acceptance of HPV vaccine. Education on immunization should also focus on medical students, as future providers of healthcare, since a recent study from Romania demonstrated a slight decline of positive perception of vaccines and vaccine usage in this group [43]. In order to better understand the main motives or attitudes for acceptance of HPV vaccine with the goal of increasing uptake of the HPV vaccine, the additional research is necessary, also among the healthcare professions. Recent study underlined several community level barriers which might impact the HPV vaccination among young girls, and importance of the involvement of general community health workers and teachers, school and health facility stakeholders, especially in low-resource settings [44]. Until then, it is crucial to implement education programs regarding the HPV vaccination and establish public dialogs in media as well as among paediatric professionals.

## Supporting information

S1 Table Additional motives and attitudes for vaccination in two comparative groups (with and without paediatricians’ recommendation, regardless of the strongest motive). (DOCX)

S1 File Database for the analyses. (XLS)
