# Supplementary material for: Motives and attitudes of parents toward HPV vaccination: Results from the initial period of HPV vaccine rollout in Serbia
Source: PLoS One. 2023 Jul 6;18(7):e0287295. doi: 10.1371/journal.pone.0287295 (PMC10325070; doi:10.1371/journal.pone.0287295)
Supplement: S1 Table — (DOCX) [file pone.0287295.s001.docx]

| **Motives from the questionnaire (number of positive answers)** | **Total (n=436)**  **n (%)** | **With pediatricians' recommendation (n=253)**  **n (%)** | **Without pediatricians' recommendation**  **(n=183) n (%)** | **p-value^1^** |
| --- | --- | --- | --- | --- |
| Parents personal experience with genital warts | 26 (6.0) | 13 (5.1) | 13 (7.1) | 0.39 |
| Family / friends discussed genital warts problems | 19 (4.4) | 12 (4.7) | 7 (3.8) | 0.64 |
| Parents personal experience with cervical cancer | 4 (0.9) | 3 (1.2) | 1 (0.5) | 0.49 |
| There were cervical cancer patients in the family and the community | 68 (15.6) | 36 (14.2) | 32 (17.5) | 0.36 |
| Parent’s awareness of the fact that HPV vaccine protects against cancers in different localization | 401 (92.0) | 237 (93.7) | 164 (89.6) | 0.12 |
| Parental anxiety due to possible infection and cancer in the child | 315 (72.2) | 195 (77.1) | 120 (65.6) | **<0.01** |
| It is better to vaccinate a child than expose them to a potential risk of HPV infection | 371 (85.1) | 228 (90.1) | 143 (78.1) | **<0.01** |
| The vaccine has been used in high-income countries for a long time, so I want my child to receive the vaccine as well | 259 (59.4) | 172 (68.0) | 87 (47.5) | **<0.01** |
| Because the vaccine is free of charge | 105 (24.1) | 69 (27.3) | 36 (19.7) | 0.07 |
| Recommendation from friends and family | 209 (47.9) | 134 (53.0) | 75 (41.0) | **0.01** |
| Parents understand the importance of this vaccine from experts heard in public media | 254 (58.3) | 172 (68.0) | 82 (44.8) | **<0.01** |
| Parents obtained information on the internet about the importance of the vaccine | 193 (44.3) | 126 (49.8) | 67 (36.6) | **<0.01** |
| Because a condom does not absolutely protect against HPV infection | 123 (28.2) | 91 (36.0) | 32 (17.5) | **<0.01** |
| Because friends vaccinated their child | 60 (13.8) | 40 (15.8) | 20 (10.9) | 0.14 |
| My child received all obligatory vaccines, so I want him/her to receive this one as well | 187 (42.9) | 122 (48.2) | 65 (35.5) | **<0.01** |
| The parent does not state the strongest motive | 16 (3.7) | 14 (5.5) | 2 (1.1) | **0.02** |
| **Strongest motive, n (%)** | ***Pediatrician recommendation,*** 88 (20.2%) | ***Pediatrician recommendation,*** 88 (34.8%) | ***Parent’s awareness of the fact that HPV vaccine protects against cancers in different localization***  40 (21.9%) | NA |

**Supplementary table 1. Additional motives and attitudes for vaccination in two comparative groups (with and without pediatricians’ recommendation regardless of the strongest motive)**

^1^Significance levels are given in bold for p<0.05. NA=not applicable.
